# A Bibliometric Analysis of Endoscopic Sedation Research: 2001–2020

**DOI:** 10.3389/fmed.2021.775495

**Published:** 2022-01-03

**Authors:** Yi Qin, Sifan Chen, Yuanyuan Zhang, Wanfeng Liu, Yuxuan Lin, Xiaoying Chi, Xuemei Chen, Zhangjie Yu, Diansan Su

**Affiliations:** Department of Anesthesiology, Renji Hospital, Shanghai Jiao Tong University School of Medicine, Shanghai, China

**Keywords:** endoscopic sedation, bibliometric analysis, hot spots, CiteSpace, VOSviewer, co-citation analysis

## Abstract

**Background and Aims:** To evaluate endoscopic sedation research and predict research hot spots both quantitatively and qualitatively using bibliometric analysis.

**Methods:** We extracted relevant publications from the Web of Science Core Collection (WoSCC) on 13 December 2020. We examined the retrieved data by bibliometric analysis (e.g., co-cited and cluster analysis, keyword co-occurrence) using the software CiteSpace and VOSviewer and the website of bibliometrics, the Online Analysis Platform of Literature Metrology (http://bibliometric.com/), to analyse and predict the trends and hot spots in this field.

**Main Results:** We identified 2,879 articles and reviews on endoscopic sedation published between 2001 and 2020. Although the overall trend is increasing, with slight fluctuation in some years, there were significant increases in 2007 and 2012. In respect of the contributions on endoscopic sedation research, the United States (US) had the greatest number of publications, and it was followed by Japan and China. In addition, collaboration network analysis revealed that the most frequent collaboration was between the US and China. Six of the top ten most prolific research institutions were located in the US. The most publications on endoscopic sedation research in the past two decades were found primarily in journals on gastroenterology and hepatology. Keyword co-occurrence and co-citation cluster analysis revealed the most popular terms relating to endoscopic sedation in the manner of cluster labels; these included patient anxiety, tolerance, ketamine, propofol, hypoxia, nursing shortage, endoscopic ultrasonography, colorectal cancer, carbon dioxide insufflation, and water exchange (WE). Keyword burst detection suggested that propofol sedation, adverse event, adenoma detection rate (ADR), hypoxemia, and obesity were newly-emergent research hot spots.

**Conclusions:** Our findings showed that hypoxia, adverse event, and ADR, along with conscious sedation and propofol sedation, have been foci of endoscopic sedation research over the past 20 years. The research focus has shifted from sedative drugs to sedative complications and endoscopy quality control, which means that there will be higher requirements and standards for sedative quality and endoscopy quality in the future.

## Introduction

Gastrointestinal endoscopy is the gold standard for early detection of gastric cancer and colorectal cancer. In the United States (US), the colorectal cancer mortality rate is by more than 50% lower than what it was two decades ago, when doctors began to systematically employ colonoscopy as a screening tool rather than as a diagnostic tool (1). Globally, a large number of gastrointestinal endoscopies are performed. Statistical analysis of the data for China in 2016 revealed that 26 million gastrointestinal endoscopies were carried out in the country (2). According to 2015 data, approximately 20 million gastrointestinal endoscopies are performed in the US on an annual basis (3). In France, more than one million esophagogastroduodenoscopies (EGDs) are performed each year (4).

Although sedation has been widely used worldwide to relieve patient anxiety and discomfort during gastrointestinal endoscopy, improve the outcome of the examination and diminish the patient's memory of the event (5), the characteristics of sedative use (including the proportion of gastrointestinal endoscopy used for sedation, sedation methods, personnel composition, equipment used, and drug selection) vary between countries. According to survey data from the US, sedation is used in more than 98% of EGDs and colonoscopies in that country (6). In Canada, sedation is used on more than 90% of colonoscopy patients (7). Conversely, in many European and Asian countries, endoscopy is usually carried out without sedation. In Germany, intravenous sedation was applied in 82% of EGDs and 91% of colonoscopies (8). In Switzerland, conscious sedation was used in 77% of EGDs and 78% of colonoscopies (9). In France, 64.7% of EGDs were performed under either sedation or general anesthesia (4). In China, 12 million of the 26 million gastrointestinal endoscopies in 2016 involved sedation (2).

The global development of science and technology has brought increased demand for endoscopy by both patients and doctors, and the demand for sedation for endoscopy has also increased. However, this has also increased both costs and cardiopulmonary complications (6, 10–14). Consequently, endoscopic sedation has undergone many changes. As yet, no scientometric study on endoscopic sedation has been reported in the Web of Science Core Collection (WoSCC) database, let alone any focus on the analysis or prediction of research hot topics or trends.

Bibliometric analysis, a widely-accepted statistical research tool for analyzing impact and evidence, has grown in popularity. Through qualitative and quantitative analysis of publications in various areas, bibliometric analysis can use literature metrology characteristics to estimate the contribution of a certain field, discover frontiers, and predict emerging trends for a specific topic. In this article, we attempt to provide a general description of quantitative and visual information in the global literature on endoscopic sedation research, identifying its emerging trends and potential hot spots from various aspects, including anesthetic drugs, anesthetic techniques, qualification of endoscopy, and adverse events through integrative analysis of relevant information from manuscripts published worldwide from 2001 to 2020. We have presented a brief discussion of endoscopic sedation research and predicted possible trends in this field over the next few years.

## Materials and Methods

We extracted the bibliographic data from the WosCC database (Clarivate Analytics, Philadelphia, PA, USA), one of the most comprehensive and authoritative databases for literature searches, using a query based on the major topics that contain the pre-defined terms in the title, abstract and keywords of the relevant manuscripts. The detailed search strategies are attached as Supplementary Material.

We applied filters to limit the search to original articles and reviews, index = science citation index expanded (SCI-EXPANDED), timespan = 2001–2020. We completed all our literature retrieval and data downloads over the course of 1 day, 13 December 2020, to reduce bias arising from frequent updates of the database.

Two reviewers (QY and CSF) independently identified all relevant manuscript information, including titles, keywords, publication years, countries/regions, institutions, authorship, and citation counts. For bibliometric analysis, we converted WosCC data to txt format and imported them into CiteSpace V5.7.R3 SE, 64bit (Drexel University, Philadelphia, PA, US), and VOSviewer 1.6.15 (Leiden University, Leiden, The Netherlands) and subsequently analyzed them both quantitatively and qualitatively. For the bibliometric analysis, WoSCC data were converted to txt format and were imported into CiteSpace V5.7.R3 SE, 64bit (Drexel University, Philadelphia, PA, USA) and the following options were used: the time-slicing was set to “2001–2020”; the number of years per slice was set to “1”; the selection criterion was set to “g-index”; and the scale factor k was set to “25”; moreover, the options “pathfinder” and “pruning the merged network” were selected in order to reduce the number of links while retaining the most salient structure; for the node type, only one option was selected at a time from “author,” “institution,” “country,” “reference,” “cited author,” and “keyword.” VOSviewer 1.6.15 (Leiden University, Leiden, The Netherlands) was used in order to create the term maps by using the following options: “Create a map based on bibliographic data,” “read data from bibliographic database files,” “type of analysis: co-occurrence,” “unit of analysis: all keywords,” “counting method: full counting,” and “minimum number of occurrence of a keyword: 25.”

We aimed to describe all literature characteristics, including countries/institutions, journals, high-cited articles, clustered networks of co-cited references, and keywords with the strongest citation bursts. In particular, we applied burst detection to keywords assigned to publications in a citation-expanded collection of articles in addition to noun phrases extracted from the articles' titles and abstracts. We will analyse the structure and dynamics of the literature of endoscopic sedation in terms of progressively synthesized networks derived from citations made by citing articles that meet various selection criteria.

## Results

### Global Publication Trend

**Figure 1:**
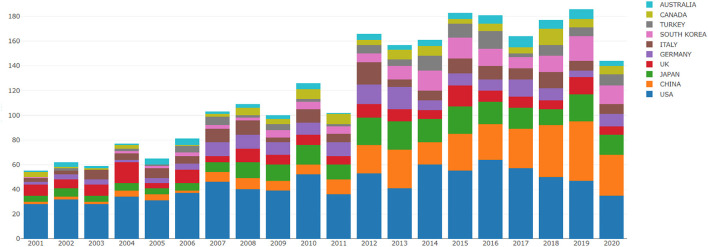
The number of annual publications and growth trends of top 10 countries/regions on Endoscopic Sedation research from 2001 to 2020.

Our literature search identified 2,879 records from 2001 to 2020. The number of papers published per year and the contribution of several countries are shown in Figure 1. Although the overall trend is increasing, with slight fluctuation in certain years, it is interesting to note that the amount of annual publications can be divided easily into three stages: stage one, from 2001 to 2006, when the average number of publications per year was between 60 and 80; stage two, from 2007 to 2011, when the average number of publications per year was between 100 and 120; and stage three, from 2012 to 2020, when there was a prominent uptick in the number of publications, rising to more than 60 for most of this period.

### Analysis of Country, Institution, and International Collaboration

**Figure 2:**
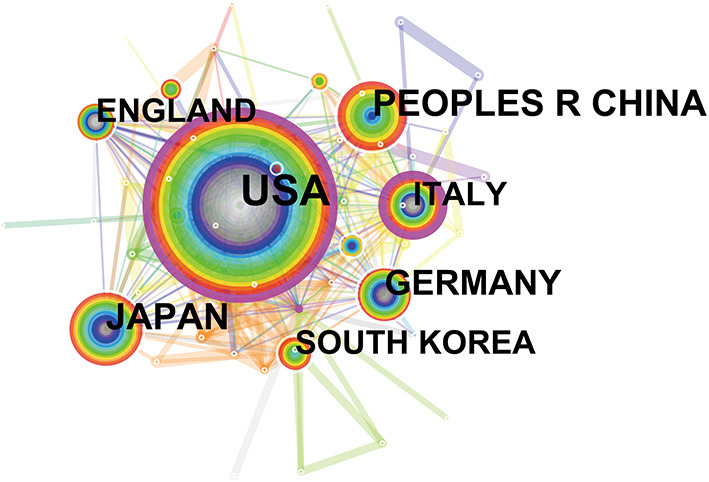
The network map of countries involved in Endoscopic Sedation research.

**Figure 3:**
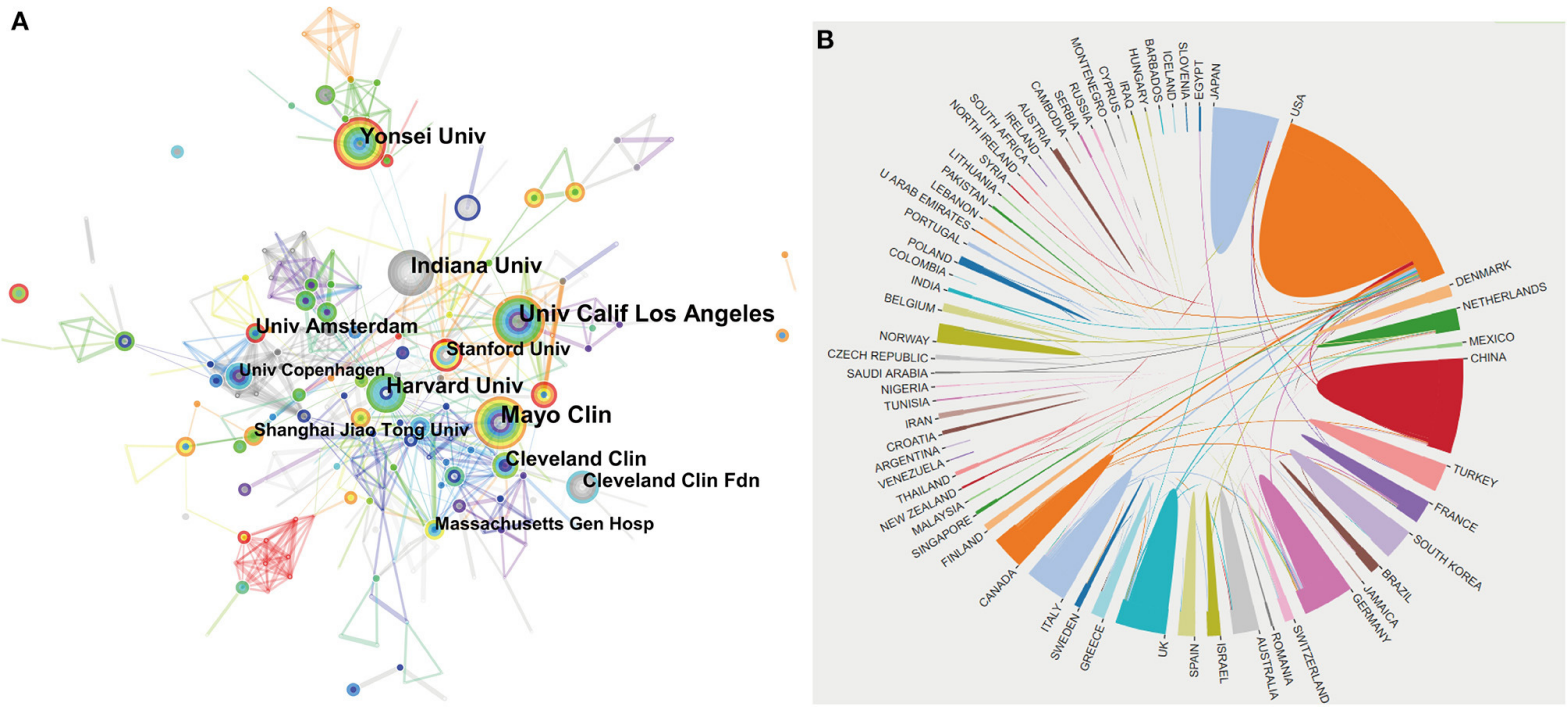
The network map of institutions involved in Endoscopic Sedation research **(A)** and collaboration between countries/regions **(B)**.

Between 2001 and 2020, the incorporated literature on endoscopic sedation was produced by at least 2,682 institutions from 85 different countries/regions. Manuscripts originated predominantly in the US, which contributed 875 papers (30.4%), followed by Japan (273; 9.5%) and China (259; 9.0%). Germany (179; 6.2%) and Italy (172; 6%) also made considerable contributions to endoscopic sedation research. Table 1 shows the detailed distribution of these countries/regions, and Figure 2 shows their co-occurrence network. Six of the top ten most prolific research institutions were located in the US, and the remaining four were in South Korea, Denmark, The Netherlands, and China, respectively. The co-occurrence network among research institutions presented a low-density map (Density = 0.0047) and most of the central indexes were below 0.10 (Figure 3A), meaning that the research groups were relatively dispersed throughout the various institutions and most institutions had a limited impact in the field. Furthermore, collaboration network analysis revealed that the most frequent collaboration occurred between the US and China, followed by the US and Canada (Figure 3B).

**Table 1:** The top 10 countries/regions and institutions contributing to publications in Endoscopic Sedation research.

**Rank**	**Country/Region**	**Article counts**	**Centrality**	**Institutions**	**Article counts**	**Centrality**	**Total number of citations**	**Average number of citations**	**Total number of first authors**	**Total number of first author citations**	**Average number of first author citations**
1	USA	875	0.27	Mayo Clin	74	0.01	289	3.91	32	180	5.63
2	JAPAN	273	0.09	Yonsei Univ	66	0.03	259	3.92	26	109	4.19
3	CHINA	259	0.03	Univ Calif Los Angeles	48	0.08	508	10.58	5	42	8.4
4	GERMANY	179	0.06	Univ Amsterdam	47	0.01	248	5.28	20	54	2.7
5	ITALY	172	0.23	Harvard Univ	46	0.12	247	5.37	10	29	2.9
6	ENGLAND	165	0.07	Cleveland Clin	44	0.03	301	6.84	16	107	6.69
7	SOUTH KOREA	153	0	Indiana Univ	39	0.02	1,200	30.77	22	483	21.95
8	TURKEY	106	0	Univ Copenhagen	38	0.00	352	9.26	13	38	2.92
9	CANADA	103	0.02	Univ Colorado	37	0.01	92	2.49	10	9	0.9
10	AUSTRALIA	91	0.05	Chinese Univ Hong Kong	36	0.02	223	6.19	14	94	6.71

### Analysis of Journals

Over the past two decades (2001–2020), 513 journals have published articles on endoscopic sedation. The 20 most active journals, as ranked by number of articles, along with impact factor (IF) Eigenfactor Score and JCR quartile as indicators of impact are listed in Table 2. These journals are more likely to accept articles on endoscopic sedation, because they have previously shown significant interest in publishing articles in this field and have produced the most publications on the related topics. The top five of these journals are *Gastrointestinal Endoscopy, Endoscopy, World Journal of Gastroenterology, Surgical Endoscopy and Other Interventional Techniques*, and *Digestive Diseases and Sciences*, together accounting for more than 18.0% of all the relevant publications. The highest IF belongs to the *American Journal of Gastroenterology* (10.17), followed by *Clinical Gastroenterology and Hepatology* (8.549), *Endoscopy* (7.341), and *Gastrointestinal Endoscopy* (6.89). The four journals mentioned above have an IF of more than five and are categorized as Q1 according to the JCR 2019 standards. Over the past two decades, the journals with the most publications on endoscopic sedation research have been mostly journals on gastroenterology and hepatology and only three out of the top 20 most active journals have been in anaesthesiology; these three are *Anesthesia and Analgesia, Pediatric Anesthesia*, and *Current Opinion in Anesthesiology*.

**Table 2:** The top 20 most active journals that published articles (sorted by count).

**Rank**	**Journal title**	**Article counts**	**Total number of citations**	**Average number of citations**	**IF (2019)**	**Eigenfactor Score**	**Quartile in category (2019)**
1	GASTROINTESTINAL ENDOSCOPY	251	10,788	42.98	6.89	0.028	Q1
2	ENDOSCOPY	155	5,395	34.81	7.341	0.015	Q1
3	WORLD JOURNAL OF GASTROENTEROLOGY	111	1,920	17.3	3.665	0.067	Q2
4	SURGICAL ENDOSCOPY AND OTHER INTERVENTIONAL TECHNIQUES	88	1,463	16.63	3.149	0.032	Q1
5	DIGESTIVE DISEASES AND SCIENCES	87	1,002	11.52	2.751	0.019	Q3
6	AMERICAN JOURNAL OF GASTROENTEROLOGY	67	4,363	65.12	10.171	0.038	Q1
7	DIGESTIVE ENDOSCOPY	65	860	13.23	4.774	0.006	Q1
8	SCANDINAVIAN JOURNAL OF GASTROENTEROLOGY	54	731	13.54	2.13	0.009	Q4
9	EUROPEAN JOURNAL OF GASTROENTEROLOGY & HEPATOLOGY	43	661	15.37	2.251	0.008	Q4
10	JOURNAL OF PEDIATRIC GASTROENTEROLOGY AND NUTRITION	40	1,066	26.65	2.937	0.016	Q3
11	DIGESTION	38	447	11.76	2.692	0.002	Q3
12	ANESTHESIA AND ANALGESIA	37	761	20.57	4.305	0.003	Q1
13	DIGESTIVE AND LIVER DISEASE	37	856	23.14	3.570	0.010	Q2
14	JOURNAL OF CLINICAL GASTROENTEROLOGY	37	615	16.62	2.973	0.009	Q3
15	JOURNAL OF GASTROENTEROLOGY AND HEPATOLOGY	37	608	16.43	3.437	0.015	Q2
16	JOURNAL OF PEDIATRIC SURGERY	35	760	21.71	1.191	0.014	Q2
17	PEDIATRIC ANESTHESIA	35	574	16.4	2.311	0.005	Q3
18	CLINICAL GASTROENTEROLOGY AND HEPATOLOGY	34	1,388	40.82	8.549	0.037	Q1
19	REVISTA ESPANOLA DE ENFERMEDADES DIGESTIVAS	33	215	6.52	2.196	0.002	Q4
20	CURRENT OPINION IN ANESTHESIOLOGY	32	345	10.78	2.276	0.004	Q3

### Analysis of Author and Co-author

**Figure 4:**
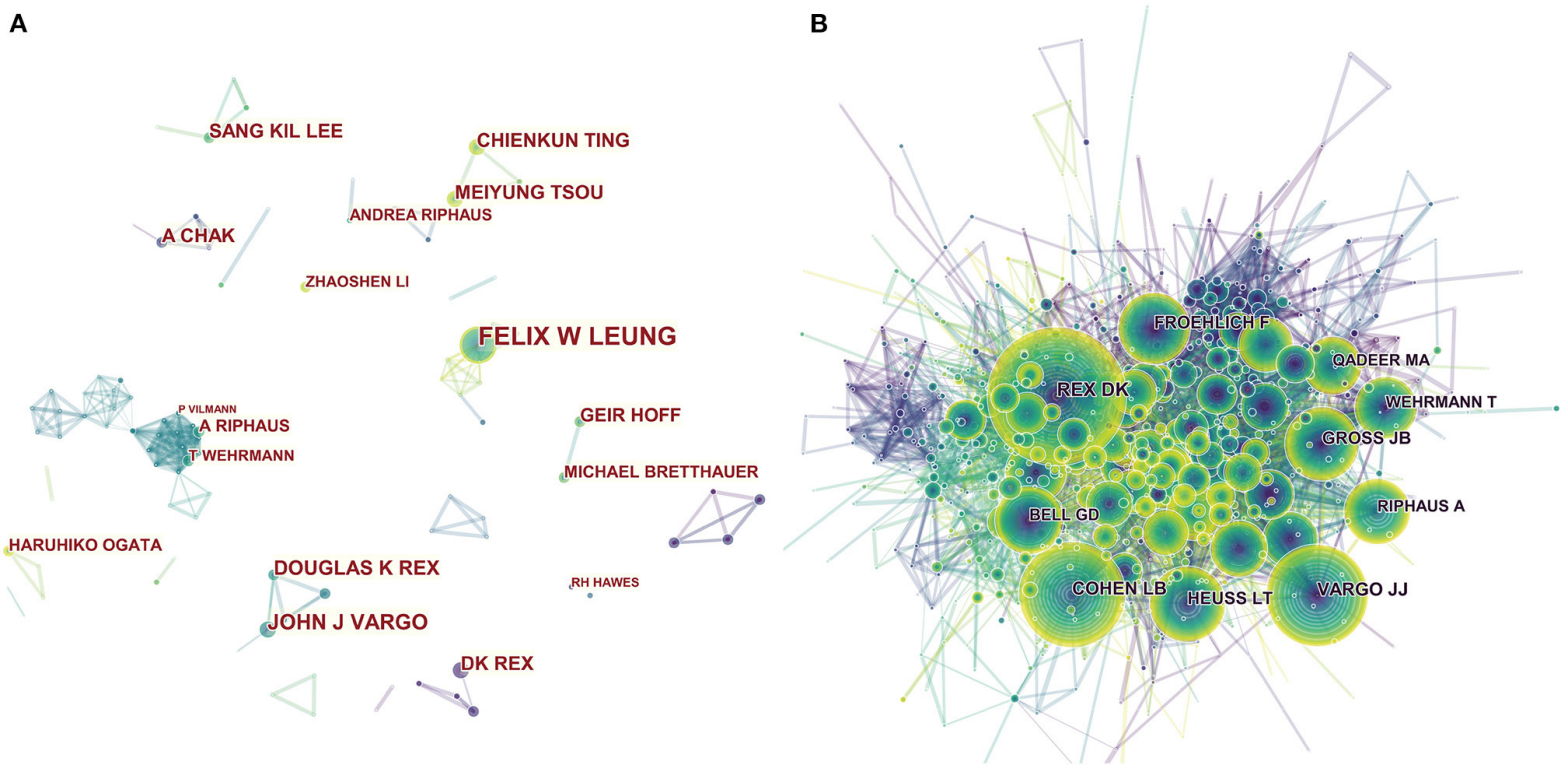
The network map of productive authors **(A)** and co-cited authors **(B)** participated in Endoscopic Sedation research.

Table 3 shows the top 10 most productive authors of the 13,282 authors in this research. Leung, Felix W, from the Department of Gastroenterological Medicine, David Geffen School of Medicine, University of California Los Angeles, US ranked first (35 articles), followed by Vargo, John J, from the Department of Gastroenterology and Hepatology, Cleveland Clinics (33 articles), and Rex, Douglas K, from the Indiana University School of Medicine (31 articles). Notably, Vargo, John J and Rex, Douglas K had more than 1,000 citations in total, suggesting that these two authors have made tremendous achievements and become authorities in endoscopic sedation research. We used CiteSpace software to visualize the networks of the citation information for authors (Figure 4A) and co-cited authors (Figure 4B). In first place was Rex, Douglas K, with 606 citations and this was followed by Cohen, Lawrence B (416), Vargo, John J (382), Gross, JB (283), and Heuss, Ludwig T (259). Of the values of centrality in the top 10 cited authors, those of four of the scholars—Rex, Douglas K (0.08), Vargo, John J (0.06), Gross, JB (0.06), and Froehlich, F (0.08)—are higher than 0.05, indicating that they have made significant contributions in the field of endoscopic sedation research and provided a crucial foundation for ongoing studies.

**Table 3:** The top 10 most productive authors and co-cited authors contributed to publications in Endoscopic Sedation research.

**Rank**	**Author**	**Article counts**	**Total number of citations**	**Average number of citations**	**First author counts**	**First author citations counts**	**Average first author citation counts**	**Corresponding author**	**Corresponding author citation counts**	**Co-cited author**	**Citation counts**	**Centrality**
1	Leung, Felix W.	35	427	12.2	15	183	12.2	18	235	Rex, Douglas K.	606	0.08
2	Vargo, John J.	33	1,104	33.45	10	369	36.9	20	675	Cohen, Lawrence B.	416	0.01
3	Rex, Douglas K.	31	1,160	37.42	7	383	54.71	21	926	Vargo, John J.	382	0.06
4	Riphaus, Andrea	26	628	24.15	14	298	21.29	12	253	Gross JB	283	0.06
5	Wehrmann, Till	26	592	22.77	6	103	17.17	9	154	Heuss, Ludwig T.	259	0.04
6	Kim, Ji Hyeong	22	48	2.18	1	0	0	3	11	Froehlich F	255	0.08
7	Hoff, Geir	22	261	11.86	5	51	10.2	4	51	Riphaus, Andrea	242	0.03
8	Bretthauer, Michael	21	255	12.14	3	113	37.67	3	113	BELL GD	207	0.04
9	Lee, Sang Kil	18	116	6.44	1	11	11	6	60	Qadeer MA	203	0.02
10	Vilmann, Peter	17	217	12.76	1	0	0	3	21	Wehrmann, Till	184	0.04

### Keyword Co-occurrence Cluster Analysis of Research Hotspots

**Figure 5:**
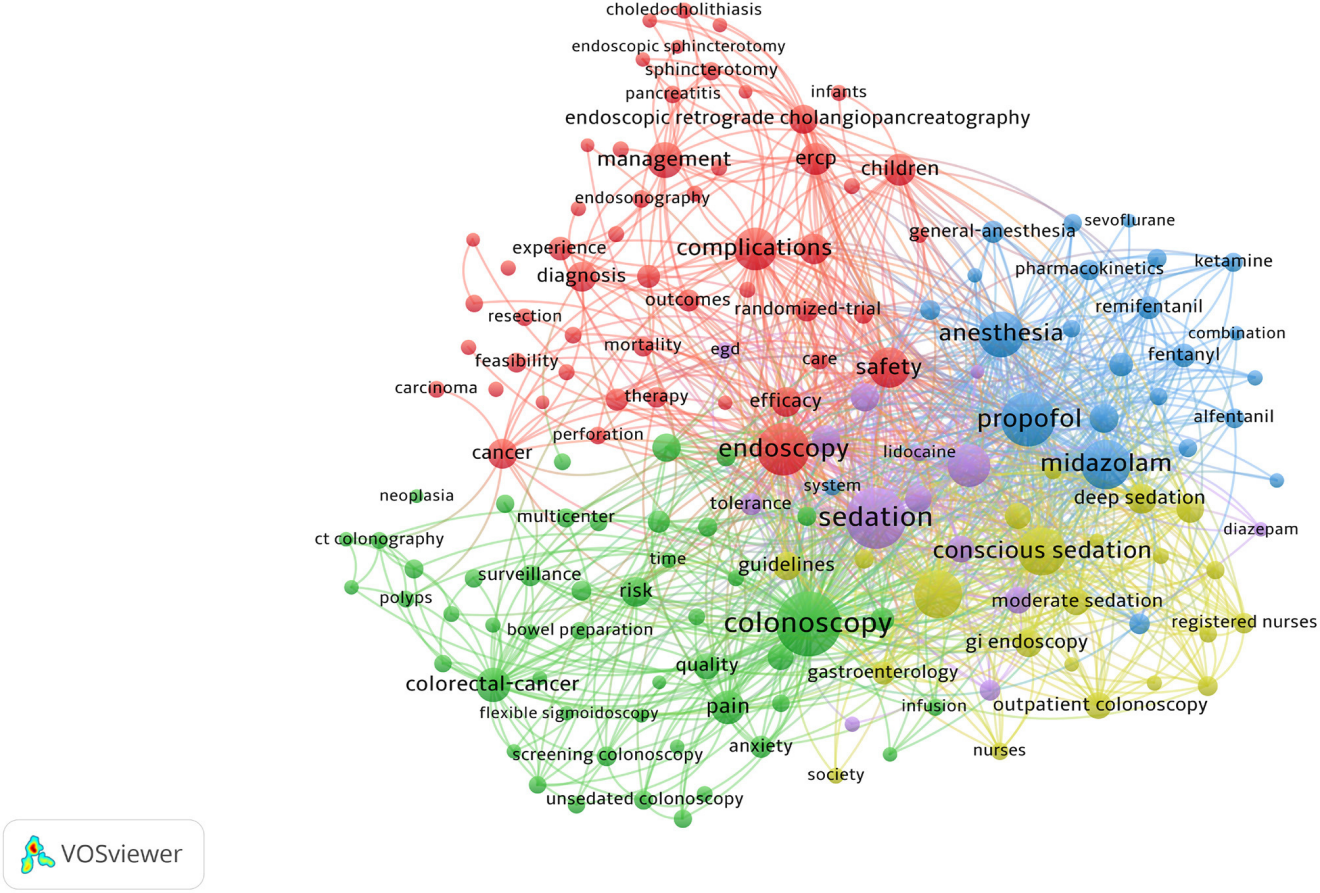
The network map of keyword clustering showed 161 keywords with a minimal occurrence of 25 times and classified into five clusters.

**Figure 6:**
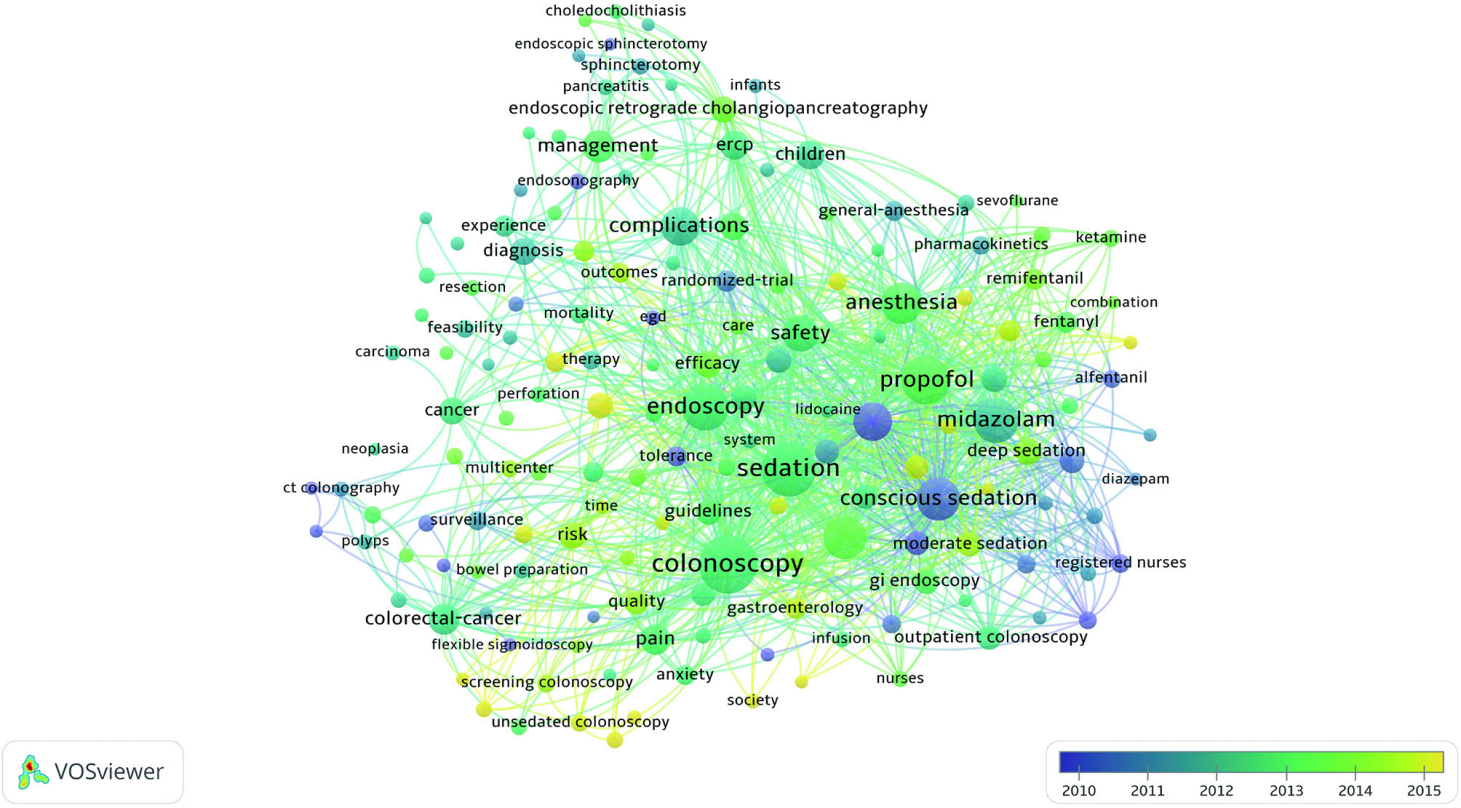
The network map of keyword clustering showed 161 keywords with a minimal occurrence of 25 times and colored differently according to the average publication year.

Keyword co-occurrence analysis provided a detailed description of hot topics covered in the endoscopic sedation research, with each article assigned author keywords and keywords plus. Through analysis of the contents of the titles and abstracts of the included manuscripts, VOSviewer identified 161 keywords that occurred a minimum of 25 times; the citation data were visualized with a bubble map. In the VOSviewer keyword co-occurrence visualization map, all keywords are grouped into clusters, with different clusters being marked in different colors. There are five clusters: endoscopy, colonoscopy, complications, anesthesia, and conscious sedation (Figure 5). In overlay visualization, there is a color bar in the bottom right-hand corner of the map, and keywords are colored differently according to the average publication year (Figure 6). For instance, “Conscious Sedation,” “Registered Nurses,” and “Alfentanil” are mainly found earlier than 2010, whereas keywords “Propofol” and “Colorectal-cancer” are more recent. Keywords such as “Unsedated Colonoscopy,” “Screening Colonoscopy,” and “Adenoma Detection Rate (ADR)” are colored yellow-green, indicating that these fields have grown in popularity in recent years and may become hotspots in the future.

### Co-cited Reference Cluster Analysis

**Figure 7:**
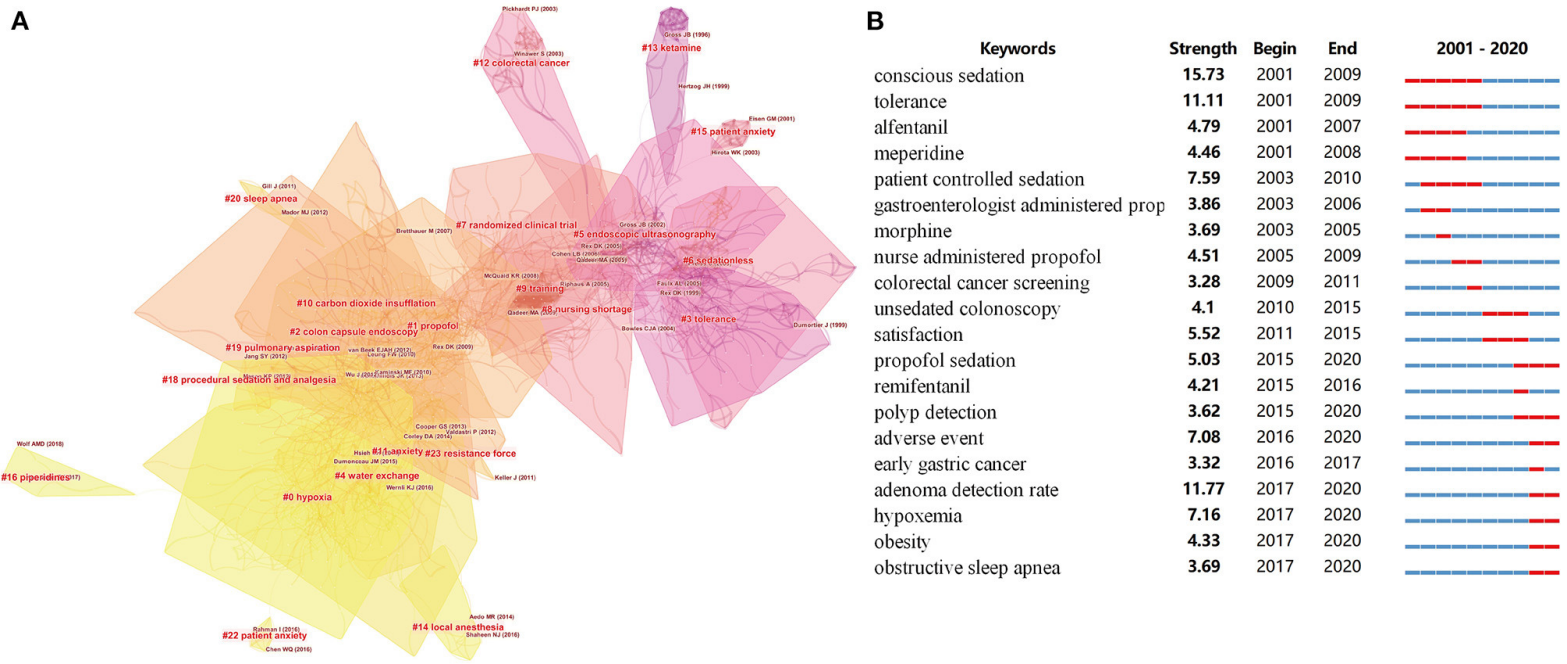
The clustered network map of co-cited references on Endoscopic Sedation. **(A)** Keywords with the strongest citation bursts in original articles on Endoscopic Sedation research between 2001 and 2020 **(B)**.

**Figure 8:**
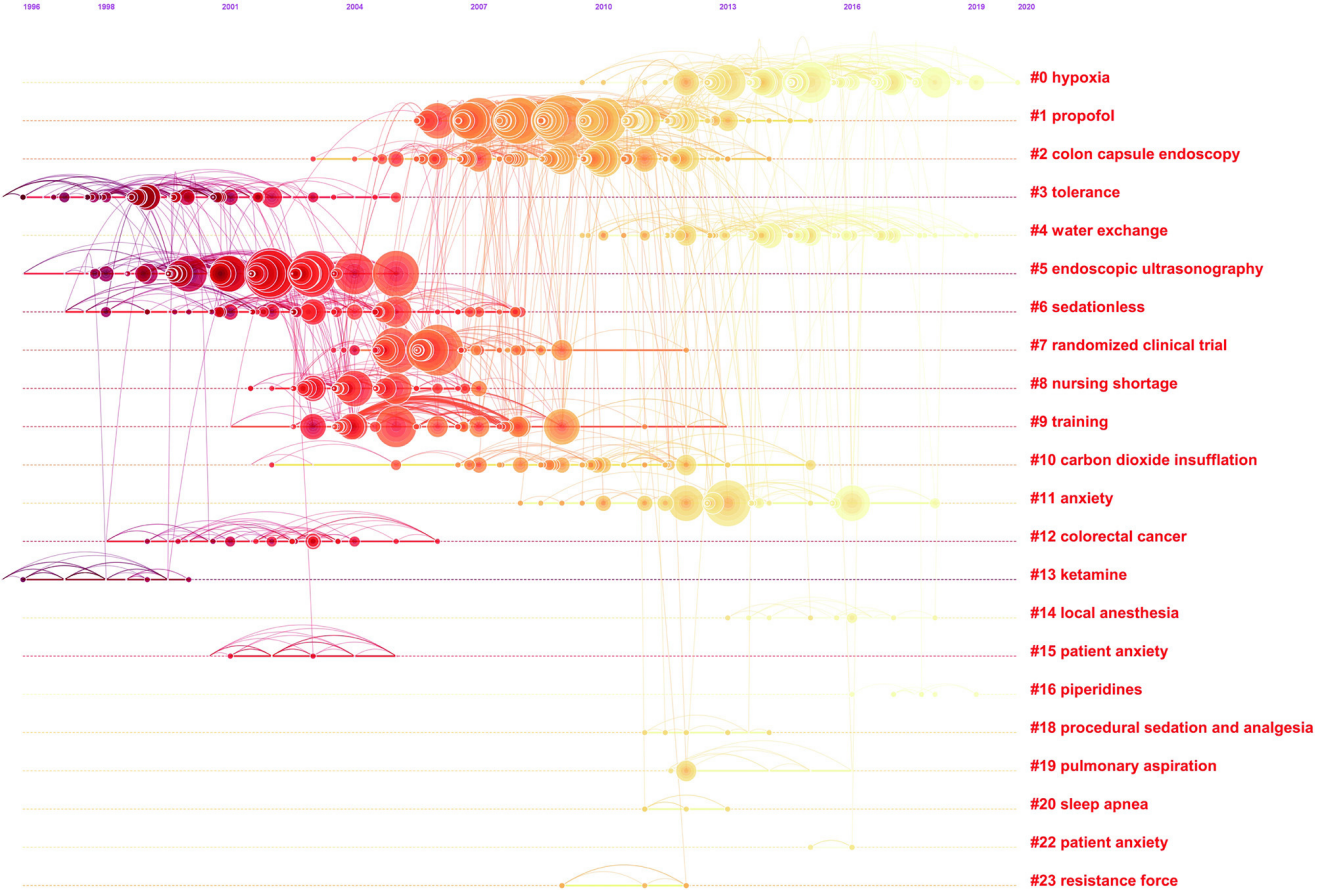
The timeline view of co-citation clusters with their cluster-labels on the right.

A co-citation network is a network of references co-cited by certain groups of publications, and a conceptual cluster is an edge that is created when a set of manuscripts are cited repeatedly together. We generated co-citation and clustered network maps from 41,134 references via CiteSpace (Figures 7, 8). We selected “Pathfinder” and “Pruning sliced networks” options to retain the most salient network structure. Visualization of co-cited references showed 1,145 nodes and 2,637 links. In this network, each node represents a cited article, and the size of each node is proportional to the total frequency of co-citation of the associated article. As shown in Figure 7A, the co-cited references were clustered into 19 major cluster labels: propofol, colon capsule endoscopy, tolerance, water exchange (WE), endoscopic ultrasonography, sedationless, randomized clinical trial, nursing shortage, training, carbon dioxide insufflation, colorectal cancer, ketamine, local anesthesia, anxiety, piperidines, procedural sedation and analgesia, pulmonary aspiration, sleep apnoea, and resistance force. A timeline view of distinct co-citation is presented in Figure 8. It shows that cluster one, i.e., propofol, had the most citation bursts and the focus of research seems to have been shifting from endoscopic ultrasonography and sedationless to hypoxia, WE and anxiety.

### Burst Detection

Burst detection revealed the emerging concepts that increased abruptly over time. We detected a keyword burst between 2001 and 2020 through analysis of the 2,889 manuscripts stemming from the WoSCC database. The timeline is depicted as a year-sliced blue line, with the period of time when a subject was observed to have a burst marked as a red section, indicating both the beginning and ending years and the duration of a citation burst. Burst patterns of keywords can reveal what was new in endoscopic sedation and the associated research foci. We excluded keywords with little or no research significance and focused particularly on keywords that were representative of the research trends in endoscopic sedation (Figure 7B). Throughout the past two decades, conscious sedation ranked first with the highest burst strength (16.0891), followed by ADR (11.77), tolerance (11.11), and patient controlled sedation (7.59). From 2001, conscious sedation, tolerance, alfentanil, and meperidine became the research foci, following by patient controlled sedation, gastroenterologist administered propofol and morphine and nurse-administered propofol. In subsequent years, some keyword bursts, such as colorectal cancer screening, unsedated coloscopy, satisfaction, remifentanil and early gastric cancer, continued for a relatively short period of time. Of note, propofol sedation, adverse event, ADR, hypoxemia, and obesity had the strongest bursts from 2015 onward, indicating that they became new research foci in endoscopic sedation.

## Discussion

We found that the focus of the literature over the past two decades has changed gradually from the choice of sedative drugs, the use of anesthetics by gastroenterologists or nurses and the degree of sedation to evaluation of endoscopic quality (such as detection rate of colorectal cancer, early gastric cancer, polyp detection rate, ADR, operating doctor satisfaction, patient experience, and patient safety) and adverse events related to sedation endoscopy and related factors.

From 2001 to 2010, the research focused on the use of anesthetics. In the first few years, the choice of drugs for moderate sedation is generally benzodiazepines combined with opioids to eliminate the patient's tension and pain. The most commonly used benzodiazepines are midazolam and diazepam. The most commonly used opioids are pethidine and fentanyl (15).

According to ASGE data, with the increasing demand for endoscopy, the number of EGDs in the US in 2006 was twice that in 1989, and the increase in colonoscopy was 3–4 times due to the increased demand for colorectal cancer screening (6, 15). The introduction of new drugs in sedative endoscopy will help improve the quality of endoscopy. The sedative effect of propofol is better than that of conventional sedation such as benzodiazepines combined with opioid analgesics in satisfying both endoscopists and patients. However, propofol sedation comes with risks, such as respiratory depression caused by increased doses, in addition to increased costs caused by the need for its use.

In the 2006 US National Survey of Sedative Endoscopy, propofol sedation during endoscopy (mostly administered by an anesthesia provider) accounted for approximately 25% of all endoscopies. Although only a quarter of endoscopists use propofol for sedation, the survey of endoscopists' satisfaction with sedative drugs revealed that endoscopists were significantly more satisfied with propofol-based sedation than with conventional sedation (6).

In the early years of the twenty-first century, several RCT studies compared propofol and traditional sedatives and found similar results; endoscopists were extremely satisfied with sedation using propofol alone, patients administered propofol had shorter recovery times (*P* < 0.001) and faster postoperative diet recovery and the use of propofol in simple endoscopic surgery can reduce complications (16–20).

A hot spot of concern from 2003 to 2009 was the use of propofol sedation by registered nurses under the supervision of an endoscopist to reduce the cost of endoscopic sedation provided by an anaesthesiologist. Although many studies have proven the safety of management by a registered nurse under the supervision of an endoscopist, it is actually not feasible in most endoscopy units in the US. Therefore, anaesthesiologists administer almost all propofol in the US (5, 12). The results in another highly cited article showed that respiratory compromise is by far the greatest and most common risk of nurse-administered propofol sedation (NAPS) (16, 18, 21–28) and that NAPS is both easier and less likely to result in respiratory depression when used for lower bowel endoscopy compared with upper gastrointestinal endoscopy. Further work is required to establish the training that will ensure that NAPS proceeds safely (29).

In recent years, article keywords have focused on the assessment of the quality of sedation endoscopy and related adverse events and related factors of sedation endoscopy.

The keywords for assessment of endoscopy quality range from colorectal cancer screening to polyp detection to early gastric cancer and to the ADR of adenomas. From 2004 to 2013, several strategies aimed at improving the quality of colonoscopy. Adenoma detection rate is used widely as a key indicator of colonoscopy quality (30). The results of the cluster analysis show that WE has been mentioned many times in recent years. Multiple randomized controlled trials show that WE colonoscopy has obvious advantages over AI colonoscopy in reducing pain and improving ADR (31–34). Due to the removal of the infused water and residual air, the time required for a WE colonoscope to absorb water and to insert the colonoscope will be longer than that of other methods (35).

In addition to the detection rate of adenoma, polyp detection rate, early tumor screening, and other indicators to assess the quality of endoscopy, the quality of colonoscopy should be evaluated in respect of the three areas of technical quality, patient experience, and patient safety to be more comprehensive. Therefore, many studies have focused on the adverse events of sedation endoscopy and the high-risk factors. The findings of a large retrospective study revealed that in patients undergoing endoscopy under conscious sedation, two-thirds of the reported unplanned adverse events were cardiopulmonary adverse events. Cardiopulmonary complications include hypoxia, hypotension, arrhythmia, and apnoea. Old age, patient ASA level, hospitalization procedures, participation of trained personnel, non-university locations, and use of supplemental oxygen during upper gastrointestinal surgery are associated with a higher number of cardiopulmonary unplanned events (36). The cluster analysis shows that the latest clusters are all around hypoxia. Over the past two decades, the drugs used for sedation endoscopy have shifted from benzodiazepines and opioids to propofol. Hypoxia is a major complication during endoscopy in patients who are sedated with propofol, and increasing attention is being paid to it.

When analyzing the high-risk factors of cardiopulmonary adverse events, older age, and poor functional status assessed by ASA classification are significant independent predictors of cardiopulmonary unplanned events (36). Other high-risk factors may be due to the generally higher ASA classification of inpatients, the lack of proficiency of trainees and non-university locations, longer examination time, and longer sedation time with, therefore, more unplanned cardiopulmonary events. The use of supplemental oxygen will delay pulse oximetry to detect apnoea. When the pulse oxygen saturation cannot accurately reflect the ventilation situation, the addition of sedative drugs causes respiratory depression, resulting in undetected hypercapnia and hypoventilation increase and, again, more unplanned cardiopulmonary events.

The results of a randomized study of more than 500 patients undergoing deep sedation colonoscopy showed that patients undergoing capnography monitoring had a significantly lower incidence of transient hypoxemia than did patients undergoing standard monitoring (37). In this study, multivariate analysis revealed the independent risk factors for hypoxemia to be age, high body mass index, history of sleep apnoea, and increased sedative dose.

Obesity is related to sleep apnoea, and they are both high-risk factors for hypoxemia. Propofol, as a commonly used drug for endoscopic sedation, may increase the likelihood of the airway anatomy causing hypoxemia due to obstruction of the muscle tension of the upper airway (38). Many studies have shown that there is a higher incidence of hypoxemia among obese patients during sedation endoscopy (39, 40). Therefore, studies are exploring different measures to reduce the incidence of hypoxia in obese patients during sedation. Two randomized controlled trials showed that supraglottic jet oxygenation (41) and high-flow nasal cannula (HFNC) supportive oxygen therapy can prevent the occurrence of American Society of Anesthesiologists grade I–II hypoxia and severe hypoxia in patients undergoing selective gastroscopy under propofol sedation. High-flow nasal cannula significantly reduced the incidence of hypoxia and severe hypoxia from 8.4 to 0% (*P* < 0.001) and 0.6 to 0% (*P* = 0.03) (42). However, the findings of a study of whether high-flow oxygen inhalation can improve sedation hypoxemia in obese patients indicated that at similar FiO_2_, HFNC did not differ significantly from standard nasal cannula in preventing arterial oxygen desaturation in morbidly obese patients undergoing propofol sedation for colonoscopy (43). Therefore, one of the current challenges of sedation endoscopy is finding a measure to improve hypoxemia in obese patients during sedation endoscopy.

Compared with traditional reviews, an analysis based on bibliometric tools (such as CiteSpace and VOSviewer) provides a better insight into the evolving research foci and trends, and this type of data analysis is comparatively more comprehensive and objective. But it comes with certain limitations. According to our inclusion criteria, only English documents were enrolled in our present analysis, so some important non-English documents might have been excluded from our analysis. Moreover, we only analyzed the documents indexed in the WoSCC database, due to the limitation of the CiteSpace software. Although most of the research manuscripts on endoscopic sedation were indexed in the WoSCC database, some other databases such as PubMed and Scopus might ensure a better representation of the available academic outputs in this field. Therefore, future work should expand the research base in order for the latter to include non-English works as well as works from other databases, and to include the latest publications that are likely to be overlooked by citation-based indicators.

In conclusion, we found that in the past 20 years, the foci of research on sedative endoscopy have ranged from the selection and use of sedative drugs to the evaluation of the quality of endoscopy to the adverse events and causes of sedative endoscopy. Our study provides clinicians with future research directions, focusing more on how to use new technologies (such as the HFNC) in order to reduce the incidence of adverse events during sedation endoscopy in patients with advanced age, obesity and/or ASA grade 3 or higher.

## Data Availability Statement

The original contributions presented in the study are included in the article/Supplementary Material, further inquiries can be directed to the corresponding author/s.

## Author Contributions

YQ and DS: study conception. YQ, XChe, and DS: study design. YZ, YL, WL, and XChi: study conduct. SC and ZY: data analysis. YQ, SC, and DS: data interpretation. YQ and SC: drafting of the manuscript. YQ, SC, YZ, YL, WL, XChi, XChe, ZY, and DS: critical revision of the manuscript for important intellectual content. All authors contributed to the article and approved the submitted version.

## Funding

This study was supported by grants from the National Natural Science Foundation of China (Nos. 81771133, 81970995, and 81874371), Shanghai Shenkang Hospital Development Center Founding (SHDC12017X11), Renji Hospital Clinical Innovation Foundation (PYII20-09, PYI20-01), Shanghai municipal Education Commission-Gaofeng Clinical Medicine Support (20191903), and State Key Laboratory of Neuroscience (SKLN-201803). The funders had no role in the analyses and interpretation of the results or writing of the manuscript.

## Conflict of Interest

The authors declare that the research was conducted in the absence of any commercial or financial relationships that could be construed as a potential conflict of interest.

## Publisher's Note

All claims expressed in this article are solely those of the authors and do not necessarily represent those of their affiliated organizations, or those of the publisher, the editors and the reviewers. Any product that may be evaluated in this article, or claim that may be made by its manufacturer, is not guaranteed or endorsed by the publisher.

## Supplementary Material

Supplementary Material 1 Search strategy for this study.

## Abbreviations

ADR: adenoma detection rate
AI: air insufflation
ASA: American Society of Anesthesiologists
ASGE: American Society for Gastrointestinal Endoscopy
CSF: sifan chen
e.g.: exempli gratia
EGDs: esophagogastroduodenoscopies
FiO_2_: fraction of inspired oxygen
HFNC: high-flow nasal cannula
i.e.: id est
IF: impact factor
JCR: journal citation reports
NAPS: nurse-administered propofol sedation
QY: Yi Qin
RCT: randomized controlled trial
SCI-EXPANDED: science citation index expanded
US: United States
WE: water exchange
WosCC: Web of Science Core Collection.
