# Supplementary material for: A Bibliometric Analysis of Endoscopic Sedation Research: 2001–2020
Source: Front Med (Lausanne). 2022 Jan 3;8:775495. doi: 10.3389/fmed.2021.775495 (PMC8761812; doi:10.3389/fmed.2021.775495)
Supplement: Supplementary Material 1 — Search strategy for this study. [file Data_Sheet_1.pdf]

Supplemental Data1

Science Citation Index Expanded (SCI-EXPANDED)

TS=(((“Cholangiopancreatography , Endoscopic Retrograde”) OR (“Retrograde Cholangiopancreatography, Endoscopic”) OR (“Cholangiopancreatographies, Endoscopic Retrograde”) OR (“Endoscopic Retrograde Cholangiopancreatographies”) OR (“Retrograde Cholangiopancreatographies, Endoscopic”) OR (“Endoscopic Retrograde Cholangiopancreatography”) OR (“ERCP”) OR (“Endoscopy , Gastrointestinal”) OR (“Endoscopies, Gastrointestinal”) OR (“Gastrointestinal Endoscopies”) OR (“Gastrointestinal Endoscopy”) OR (“Surgical Procedures, Endoscopic Gastrointestinal”) OR (“Endoscopic Gastrointestinal Surgical Procedures”) OR (“Gastrointestinal Endoscopic Surgical Procedures”) OR (“Surgical Procedure, Endoscopic Gastrointestinal”) OR (“Procedure, Gastrointestinal Endoscopic Surgical”) OR (“Procedures, Gastrointestinal Endoscopic Surgical”) OR (“Procedures, Gastrointestinal Endoscopic Surgical”) OR (“Endoscopic Gastrointestinal Surgery”) OR (“Endoscopic Gastrointestinal Surgeries”) OR (“Gastrointestinal Surgeries, Endoscopic”) OR (“Gastrointestinal Surgery, Endoscopic”) OR (“Surgeries, Endoscopic Gastrointestinal”) OR (“Surgery, Endoscopic Gastrointestinal”) OR (“Procedure, Endoscopic Gastrointestinal, Surgical”) OR (“Balloon Enteroscopy”) OR (“Balloon Enteroscopies”) OR (“Enteroscopies, Balloon”) OR (“Enteroscopy, Balloon”) OR (“Colonoscopy”) OR (“Colonoscopies”) OR (“Colonoscopic Surgical Procedures”) OR (“Colonoscopic Surgical Procedure”) OR (“Procedure, Colonoscopic Surgical”) OR (“Procedures, Colonoscopic Surgical”) OR (“Surgical Procedure, Colonoscopic”) OR (“Surgery, Colonoscopic”) OR (“Surgical Procedures, Colonoscopic”) OR (“Colonoscopic Surgery”) OR (“Colonoscopic Surgeries”) OR (“Surgeries, Colonoscopic”) OR (“Sigmoidoscopy”) OR (“Sigmoidoscopies”) OR (“Proctosigmoidoscopy”) OR (“Proctosigmoidoscopies”) OR (“Sigmoidoscopic Surgical Procedures”) OR (“Procedure, Sigmoidoscopic Surgical”) OR (“Procedures, Sigmoidoscopic Surgical”) OR (“Sigmoidoscopic Surgical Procedure”) OR (“Surgical Procedure, Sigmoidoscopic”) OR (“Surgery, Sigmoidoscopic”) OR (“Surgical Procedures, Sigmoidoscopic”) OR (“Sigmoidoscopic Surgery”) OR (“Sigmoidoscopic Surgeries”) OR (“Surgeries, Sigmoidoscopic”) OR (“Duodenoscopy”) OR (“Duodenoscopies”) OR (“Duodenoscopic Surgical Procedures”) OR (“Duodenoscopic Surgical Procedure”) OR (“Procedure, Duodenoscopic Surgical”) OR (“Procedures, Duodenoscopic Surgical”) OR (“Surgical Procedure, Duodenoscopic”) OR (“Surgery, Duodenoscopic”) OR (“Surgical Procedures, Duodenoscopic”) OR (“Duodenoscopic Surgery”) OR (“Duodenoscopic Surgeries”) OR (“Surgeries, Duodenoscopic”) OR (“Endoscopic Mucosal Resection”) OR (“Endoscopic Mucosal Resections”) OR (“Mucosal Resection, Endoscopic”) OR (“Mucosal Resections, Endoscopic”) OR (“Resection, Endoscopic Mucosal”) OR (“Resections, Endoscopic Mucosal”) OR (“Strip Biopsy”) OR (“Biopsies, Strip”) OR (“Biopsy, Strip”) OR (“Strip Biopsies”) OR (“Endoscopic Mucous Membrane Resection”) OR (“Endoscopic Submucosal Dissection”) OR (“Dissection, Endoscopic Submucosal”) OR (“Dissections, Endoscopic Submucosal”) OR (“Endoscopic Submucosal Dissections”) OR (“Submucosal Dissection, Endoscopic”) OR (“Submucosal Dissections, Endoscopic”) OR (“Esophagoscopy”) OR (“Esophagoscopies”) OR (“Esophagoscopic Surgical Procedures”) OR (“Esophagoscopic

Surgical Procedure”) OR (“Procedure, Esophagoscopic Surgical”) OR (“Procedures, Esophagoscopic Surgical”) OR (“Surgical Procedure, Esophagoscopic”) OR (“Surgery, Esophagoscopic”) OR (“Surgical Procedures, Esophagoscopic”) OR (“Esophagoscopic Surgery”) OR (“Esophagoscopic Surgeries”) OR (“Surgeries, Esophagoscopic”) OR (“Gastroscopy”) OR (“Gastroscopies”) OR (“Gastroscopic Surgical Procedures”) OR (“Gastroscopic Surgical Procedure”) OR (“Procedure, Gastroscopic Surgical”) OR (“Procedures, Gastroscopic Surgical”) OR (“Surgical Procedure, Gastroscopic”) OR (“Surgery, Gastroscopic”) OR (“Surgical Procedures, Gastroscopic”) OR (“Gastroscopic Surgery”) OR (“Gastroscopic Surgeries”) OR (“Surgeries, Gastroscopic”) OR (“Proctoscopy”) OR (“Proctoscopies”) OR (“Proctoscopic Surgical Procedures”) OR (“Procedure, Proctoscopic Surgical”) OR (“Procedures, Proctoscopic Surgical”) OR (“Proctoscopic Surgical Procedure”) OR (“Surgical Procedure, Proctoscopic”) OR (“Surgery, Proctoscopic”) OR (“Proctoscopic Surgeries”) OR (“Surgeries, Proctoscopic”) OR (“Surgical Procedures, Proctoscopic”) OR (“Proctoscopic Surgery”) OR (esophagogastroduodenoscopy) OR (“GI endoscopy”) OR (“Endoscopes, Gastrointestinal”) OR (“Endoscope, Gastrointestinal”) OR (“Gastrointestinal Endoscope”) OR (“Gastrointestinal Endoscopes”) OR (“Capsule Endoscopes”) OR (“Capsule Endoscope”) OR (“Endoscope, Capsule”) OR (“Endoscopes, Capsule”) OR (“Video Capsule Endoscopes”) OR (“Capsule Endoscope, Video”) OR (“Capsule Endoscopes, Video”) OR (“Endoscope, Video Capsule”) OR (“Endoscopes, Video Capsule”) OR (“Video Capsule Endoscope”) OR (“Colonoscopes”) OR (“Colonoscope”) OR (“Sigmoidoscopes”) OR (“Sigmoidoscope”) OR (“Duodenoscopes”) OR (“Duodenoscope”) OR (“Esophagoscopes”) OR (“Esophagoscope”) OR (“Gastroscopes”) OR (“Gastroscope”) OR (“Proctoscopes”) OR (“Endosonography”) OR (“Endosonographies”) OR (“Endoscopy, Echo”) OR (“Echo Endoscopies”) OR (“Endoscopies, Echo”) OR (“Ultrasonic Endoscopy”) OR (“Echo-Endoscopy”) OR (“Echo Endoscopy”) OR (“Echo-Endoscopies”) OR (“Endoscopy, Ultrasonic”) OR (“Endoscopies, Ultrasonic”) OR (“Ultrasonic Endoscopies”) OR (“Ultrasonography, Endoscopic”) OR (“Endoscopic Ultrasonography”) OR (“Endoscopic Ultrasonographies”) OR (“Ultrasonographies, Endoscopic”) OR (“Transanal Endoscopic Surgery ”) OR (“Endoscopic Surgeries, Transanal”) OR (“Endoscopic Surgery, Transanal”) OR (“Surgeries, Transanal Endoscopic”) OR (“Surgery, Transanal Endoscopic”) OR (“Transanal Endoscopic Surgeries”) OR (“Transanal Endoscopic Surgical Procedures”) OR (“Transanal Minimally Invasive Surgery”) OR (“TAMIS”) OR (“Transanal Endoscopic Microsurgery”) OR (“Endoscopic Microsurgeries, Transanal”) OR (“Endoscopic Microsurgery, Transanal”) OR (“Microsurgeries, Transanal Endoscopic”) OR (“Microsurgery, Transanal Endoscopic”) OR (“Transanal Endoscopic Microsurgeries”) OR (“Pyloromyotomy”) OR (“ Peroral endoscopic myotomy”) OR (“peroralendoscopicmyotomy”) OR (“Pyloromyotomies”) OR (“Ramstedt Operation”) OR (“Fredet-Ramstedt Operation”) OR (“Fredet Ramstedt Operation”) OR (“Gastric Peroral Endoscopic Pyloromyotomy”) OR (“POEM Procedure”) OR (“POEM Procedures”) OR (“G-POEM”) OR (“Gastric Per-oral Endoscopic Pyloromyotomy”) OR (“Gastric Per oral Endoscopic Pyloromyotomy”) OR (“Sphincterotomy, Endoscopic”) OR (“Endoscopic Sphincterotomy”) OR (“Endoscopic Sphincterotomies”) OR (“Sphincterotomies, Endoscopic”) OR (“Endoscopic Papillotomy”) OR (“Endoscopic Biliary Sphincterotomy”) OR (“Biliary Sphincterotomies, Endoscopic”) OR (“Biliary Sphincterotomy, Endoscopic”) OR (“Endoscopic Biliary Sphincterotomies”) OR

(“Sphincterotomies, Endoscopic Biliary”) OR (“Sphincterotomy, Endoscopic Biliary”) OR (“Papillotomy, Endoscopic”) OR (“Endoscopic Papillotomies”) OR (“Papillotomies, Endoscopic”) OR (esophagogastroduodenoscopies) OR (EGD) OR (EGDs)) AND ((“Conscious Sedation”) OR (“Sedation, Moderate”) OR (“Moderate Sedation”) OR (“Sedation, Conscious”) OR (“Painless”) OR (sedation) OR (sedative) OR (sedated) OR (anesthesia) OR (anaesthesia) OR (anesthesias) OR (anesthetics)))

Languages=English

Document type=Article OR Review

Timespan=2001-2020
